# Trends of Body Mass Index changes among adults on antiretroviral therapy in Northwest Ethiopia: a longitudinal data analysis

**DOI:** 10.1038/s41598-024-53701-0

**Published:** 2024-03-04

**Authors:** Berihun Bantie, Natnael Atnafu Gebeyehu, Getachew Asmare Adella, Gizachew Ambaw Kassie, Misganaw Asmamaw Mengstie, Endeshaw Chekol Abebe, Mohammed Abdu Seid, Molalegn Mesele Gesese, Kirubel Dagnaw Tegegne, Denekew Tenaw Anley, Melkamu Aderajew Zemene, Anteneh Mengist Dessie, Sefineh Fenta Feleke, Tadesse Asmamaw Dejenie, Yenealem Solomon Kebede, Ermias Sisay Chanie, Gashaw Kerebeh, Wubet Alebachew Bayih, Natnael Moges

**Affiliations:** 1 Department of Comprehensive Nursing, College of Health Sciences, Debre Tabor University, Debre Tabor, Ethiopia; 2 Department of Midwifery, College of Medicine and Health Science, Wolaita Sodo University, Wolaita, Ethiopia; 3 Department of Reproductive Health and Nutrition, School of Public Health, Woliata Sodo University, Sodo, Ethiopia; 4 Department of Epidemiology and Biostatistics, School of Public Health, Woliata Sodo University, Sodo, Ethiopia; 5 Department of Biochemistry, College of Health Sciences, Debre Tabor University, Debre Tabor, Ethiopia; 6 Unit of Physiology, Department of Biomedical Science, College of Health Science, Debre Tabor University, Debre Tabor, Ethiopia; 7 Department of Nursing, College of Medicine and Health Science, Wollo University, Dessie, Ethiopia; 8 Department of Public Health, College of Health Sciences, Debre Tabor University, Debre Tabor, Ethiopia; 9 Department of Public Health, College of Health Sciences, Woldia University, Woldia, Ethiopia; 10 Department of Medical Biochemistry, College of Medicine and Health Sciences, University of Gondar, Gondar, Ethiopia; 11 Department of Medical Laboratory Science, College of Health Sciences, Debre Tabor University, Debre Tabor, Ethiopia; 12 Department of Pediatrics and Child Health Nursing, College of Health Sciences, Debre Tabor University, Debre Tabor, Ethiopia

**Keywords:** Diseases, Health care, Medical research

## Abstract

Nutritional status is considered a major diagnostic and prognostic indicator of HIV/AIDS in adults. In this aspect, current HIV-treatment guidelines, particularly in low-income countries, recommend the regular monitoring of body mass index (BMI) to determine patients' clinical response to antiretroviral therapy (ART). However, data regarding the change in BMI status of HIV-positive adults on ART following the implementation of the test and treat strategy were limited in Ethiopia. Hence, this study is aimed at investigating the trends of BMI change over time and its associated factors among HIV-positive adults in Northwest Ethiopia. A retrospective longitudinal study was conducted among 404 randomly selected HIV-positive adults receiving ART in Felegehiwot Comprehensive Specialized Hospital (FHCSH), Northern Ethiopia. Data were extracted from the medical record charts of study participants, entered into Epi-data 4.6 software, and exported to Stata 14.2 software for analysis. A generalized estimating equation (GEE) model was fitted to determine the change in BMI status over time and its predictors in HIV-positive adults. The level of significance was declared at a p-value of < 0.05. More than half (201, or 51.73%) of the total 404 participants were female. In the cohort, both the baseline and follow-up mean body mass index levels of the participants fell in the normal range and increased from 20.34 (standard deviation/SD ± 2.8) to 21.41 (SD ± 3.13). The individual profile plots of 50 participants indicated that there is considerable variability in weight change across individuals. Duration of ART follow-up (β = 0.203, 95% confidence interval (CI) 0.16 to 0.24), unemployment (β = − 0.96, 95% CI 1.67 to − 0.25), WHO stage III/IV HIV disease (β = − 0.92, 95% CI − 1.57 to − 0.35),and Tenofovir/Lamivudine/Dolutegravir (TDF/3TC/DTG)ART regimen (β = 0.95, 95% CI 0.32 to 1.57) were identified as significant predictors of change in the BMI status of participants. Likewise, the interaction of TDF/3TC/DTG ART regimen * follow-up duration (β = 2.16, 95% CI 1.84 to 2.84), WHO stage III/IV clinical disease * follow-up duration (β = − 1.43, 95% CI − 1.71 to − 1.15) and TB/HIV co-infection * follow-up duration (β = 1.89, 95% CI 1.57 to 2.87) significantly affects the trend in BMI change status of HIV-positive adults. In this study, the BMI status of HIV-positive adults receiving ART increased with a linear trend. Unemployment, stage III/IV HIV diseases, and Tenofovir/Lamivudine/Efavirenz (TDF/3TC/EFV) ART-drug regimen decreases the mean BMI status of HIV-positive adults. Special consideration and strict follow-up need to be given to those individuals with advanced HIV/AIDS diseases and other identified risk group.

## Introduction

Malnutrition, particularly undernutrition, is one of the most common public health problems among people living with HIV/AIDS, particularly in low- and middle-income countries, including Ethiopia^1–3^. They are closely related terms, and their relationship is bidirectional^1,3–6^. HIV is one of the major causes of malnutrition in adults due to its effects on loss of appetite, nausea and vomiting, diarrhea, opportunistic infections, malabsorption of essential nutrients, and the increasing metabolic needs of the body^1,4,7–10^. Compared to healthy individuals, adult HIV/AIDS-positive patients require 10% more energy consumption when asymptomatic, 20–30% more when symptomatic, and 30% more when recovering^4,6^. In this context, in Sub-Saharan African (SSA) countries, nearly one-fourth (23.7%) of peoples living with HIV/AIDS are undernourished^11^. Similarly, the pooled prevalence of under nutrition among HIV-positive adults in Ethiopia is estimated to be 26%^12^, with a wide variation across regions.

On the other hand, under nutrition weakens the body’s immune system and makes individuals vulnerable to infections, which all accelerate the progression of HIV infection to the AIDS stage^9,13,14^. In this context, nutritional status is considered as a major diagnostic and prognostic indicator of HIV/AIDS. The World Health Organization (WHO) clinical staging of HIV/AIDS patients is also highly dependent on the nutritional status of HIV-positive patients^15^. For instance, those HIV-positive adults with moderate unexplained weight loss (5–10%) will be classified as stage II; patients with unexplained severe weight loss (> 10%) are classified as stage III; and those with HIV wasting syndrome are classified as stage IV clinical diseases. Besides, HIV-associated weight loss and a low body mass index (BMI) are found to be independent indicators of poor treatment outcomes, including death^16^. The risk of death among HIV-positive patients who were malnourished was nearly twice as high as that of their counterparts^3,17–19^. In light of this, in a setting with low socioeconomic status where tracking the virologic and immunologic response of HIV-positive patients is challenging, routine follow-up of BMI changes is used as a key indicator to monitor the progress of patients on ART.

Nevertheless, HIV-positive individuals are expected to gain weight after initiation of ART due to the reversal of gastrointestinal function, the return of appetite, and the decreased incidence of opportunistic infections^20–23^. Therefore, to boost the effect of ART on reducing under nutrition and HIV-related mortality, the WHO launches initiating ART as early as possible (test and treat strategy), including same-day initiation^24^. The universal test- and- treat strategy is presumed to be more beneficial in lowering the viral load and revitalizing the immune system, thereby improving the patient's quality of life and nutritional condition. However, not all patients on ART experience optimal weight changes; some lose weight, others maintain their weight, and others gain too much weight. For instance, about 20% and 3% of HIV-positive adults on ART in Ethiopia were undernourished (BMI < 18.5 kg/m^2^) and overweight (BMI > 25.0 kg/m^2^), respectively^25,26^. The double burden malnutrition (under nutrition and overweight) directly poses a substantial threat to the success of HIV programs in Ethiopia, and if it is not properly addressed, it may hinder the progress towards reaching HIV program targets.

To maintain optimal nutritional status and quality of life in HIV-positive adults, nutritional policies and strategies were supplemented with HIV-management guidelines both globally and in Ethiopia^27,28^. In line with this, current HIV-treatment guidelines advocate that health professionals strictly monitor the BMI change of all HIV-positive individuals and take appropriate action. In Ethiopia, nutritional assessment and interventions were given top priority because of the increased burden of both undernutrition and overweight^28^. In this regard, the weight and height of all HIV-positive patients were measured on each follow-up visit. Despite the fact that BMI is considered the most effective indicator to monitor a patient's clinical response, data on the change in BMI levels and its predictors among HIV-positive adults on ART in Ethiopia is limited. Hence, this study is targeted to provide information about the BMI change trends and their associated factors among adults receiving ART following the implementation of the universal test and treat strategy in Ethiopia. The findings of this study are important for health professionals, policymakers, and program managers to improve the quality of life of HIV-positive patients through enhancing their nutritional status.

## Methods and materials

### Study setting, design, and period

An institution-based retrospective longitudinal study was conducted among adults receiving ART at Felegehiwot Comprehensive Specialized Hospital (FHCSH), northwest Ethiopia. FHCSH is one the most earliest hospital in Amhara region, located 565 km northwest of Addis Abeba, the capital city of Ethiopia. The hospital carries the highest number of HIV-patient load, followed by Dessie comprehensive specialized hospital and Gondar University comprehensive specialized hospital. To date, there are about 6645 HIV-positive patients receiving care at the hospital. Indeed, the hospital is the main site for the viral load monitoring of patients from nearby ART centers and zones.

### Population

All HIV-positive individuals aged ≥ 15 who were enrolled on ART between January 2017 and December 2020, after the implementation of the universal test and treat strategy, were the study population. All HIV-positive individuals aged ≥ 15 who were newly initiated to ART between January 2017 and December 2020 and had at least two monthly ART follow-up visits were included. Pregnant women and individuals with incomplete records of age were excluded.

### Sample size and sampling procedure

The EP-INFO Version 4.3.2.1 software was used to calculate the minimum sample size for the study, taking into account the following assumptions: level of significance (5%); power of 80%; Z a/2 of 1.96; exposed to unexposed ratio: 1:1; and, from previous related studies, the proportion of outcome in the unexposed group (12%) and adjusted odds ratio (AOR) of 2.2)^29^. The final sample size for estimating BMI changes over time and predictors was 404. Computer-generated simple random sampling technique was used to select the final study participants. Initially, a list containing Medical registration Number (MRN) of eligible participants was extracted from the electronic data base of the hospital. Then, this number was exported to Microsoft excel v.13 software to randomly generate a total of 404 study participants.

### Variables and data collection procedures

The data abstraction checklist was developed after reviewing different literature and referring to the patient intake, follow-up, and monitoring formats developed by the Ministry of Health in Ethiopia. The tool included socio-demographic, immunologic, and clinical-related variables, behavior-related variables, and a longitudinal measurement of the Body Mass Index (BMI). The tool was initially tested using 10 charts to check the consistency of the data abstraction checklist and the patient medical recording system. Then, one-day training was given for both the data collectors and supervisors of the study about the overall purpose of this study, how to extract all relevant data from patient charts, and how to guarantee the confidentiality of patient information. The Body Mass Index (BMI) was obtained by dividing the weight in kilograms by the height in meters squared (kg/m^2^)^27^. The weight of the study participants was determined using a beam balance with a precision of 0.1 kg. Before weighing each participant, the calibration was done by setting the scale to zero. The respondents' heights were measured using a vertical height scale in the middle of the board, standing upright, and recorded to the nearest 0.1 cm^27^.

### Operational definitions

HIV-disclosure status is defined as revealing one’s HIV/AIDS status to another individual, and it is recorded as “yes” when one’s HIV positive status is informed to at least one individual^27^. ART drug adherence is assessed through counting the pills in each visit and is classified as good (≥ 95% of the doses taken), fair (85–94% of the doses taken), and poor (< 85% of the doses taken)^27^. The employment status of HIV-positive adults was categorized as employed (government and private organization’s) and non-employed (farmer, housewife, daily laborer, merchant, student, driver, etc.)^30,31^.

### Data processing and analysis

The data was initially coded, entered into Epi Data version 4.6.0, and exported to STATA V. 14.2 software for further statistical analysis. Descriptive statistics were computed with either mean with standard deviation (SD) or median with interquartile range (IQR) for continuous variables and frequency with percentage for categorical variables, respectively. Since the BMI is a continuous variable, exploratory data analysis of the BMI variation status of the study participants was done using individual profile plots, mean profile plots, and spaghetti plots. The spaghetti plot (with times of follow-up on the X axis and the BMI score on the Y axis) was used to see the pattern of the BMI change over time. Since the independence of observation assumption in the ordinal models is violated in repeated measurement, a Generalized Estimating Equation (GEE) model (using the stata command: xtgee depvar indepvar) that handles the independence of observation assumptions was fitted to assess the trends of BMI changes and its predictors among HIV-positive adults. Five GEE models with different correlation structures, namely exchangeable, unstructured, autoregressive, independent, and M-dependent (stationary), were fitted sequentially. The QIC (Quasi-likelihood Independence Model Criterion) value, which is an extension of the widely used Akaike information criterion (AIC), was used to select the best working correlation structure. In this regard, due to its lowest QIC value, the model with an exchangeable working correlation structure was considered the best-fitting model (Table 1). Variables with a p-value < 0.25 in the bivariable analysis were fitted into the multivariable GEE model. Finally, the coefficient with its 95% confidence interval and the corresponding p-values < 0.05 were used to identify the statistically significant predictors of the BMI change status of the participants.

**Table 1:** Model comparison using working correlation structure to estimate BMI changes over time among adults receiving ART at Felegehiwot comprehensive specialized hospital, 2023.

S. no.	Working correlation structure	QIC value
1	Exchangeable	17,992.29
2	Autoregressive (AR1)	18,007.422
3	Unstructured	1,820,012
4	Independent	17,994.712
5	Stationary (M-dependent)	180,023

*QIC* quasi-likelihood under Independence model Criterion.

### Ethical approval and consent to participate

Since the study used secondary data (by reviewing patients’ medical record) as a source of information and there is no direct involvement of the study participants, informed consent was not required for this study. The consent was waived by the Institution Review Board (IRB) of Bahirdar University with a protocol number of 058/2021. Indeed, all methods were carried out in accordance with relevant guidelines and regulations.

## Results

### Socio-demographic characteristics of the study participants

A total of 404 (100%) study participants were included in the final analysis. Of the total, more than half (211, 52.23%) were female. The median age of the study participants was 30 (IQR 39–25), and the majority of them (151, 37.38%) were between the ages of 25 and 344. Regarding the educational status, more than one-fourth (116, 28.71%) of the study participants didn’t attend any formal education sessions (Table 2).

**Table 2:** Baseline socio-demographic characteristics of HIV-positive adults receiving ART in Felegehiwot comprehensive specialized hospital, 2023 (N = 404).

Variable	Category	Frequency	%
Sex	Male	193	47.77
	Female	211	52.23
Age	Age 15–24	96	23.76
	Age 25–34	151	37.38
	Age 35–44	101	25.00
	Age > 45	56	13.86
Median age = 30, IQR (39–25)			
Educational status	Not have formal education	116	28.71
	Primary	110	27.23
	Secondary	110	27.23
	Tertiary	77	19.06
Marital status	Married	174	43.1
	Not married	230	56.9
Occupation status	Employed	123	30.45
	Non- employed	281	69.55
HIV-positive family members	Yes	179	44.31
	No	225	55.69

### Baseline and follow-up clinical characteristics of the study participants

Considering the clinical condition of the participants, nearly one-fourth (94, or 23.3%) of them had TB/HIV co-infection. The majority of the study participants had a CD4 cell count less than 200–500 cells/mm^3^ (187, 46.29%). Indeed, nearly half of them (182, or 45.05%) had WHO clinical stage III or IV diseases. Only two-thirds of HIV-positive adults (255, or 63.12%) disclosed their status to at least one person who will care for them (Table 3).

**Table 3:** Baseline and follow-up clinical characteristics of HIV-positive adults receiving ART in Felegehiwot comprehensive Hospital, 2023 (N = 404).

Variable	Category	Frequency	%
Baseline TB/HIV co-infection	Yes	94	23.27
	No	310	76.73
Presence of OIs	Yes	175	43.32
	No	229	56.68
Baseline CD4 level	> 500 cell/mm^3^	103	25.50
	200–500 cell/mm^3^	188	46.53
	< 200 cells/mm^3^	113	27.97
Baseline WHO clinical staging	WHO stage I/II	221	54.70
	WHO stage III/IV	183	45.30
Chronic diseases	Yes	41	10.15
	No	363	363
Functional status	Working	307	75.99
	Ambulatory/bedridden	97	24.01
IPT utilization	Yes	226	55.94
	No	178	44.06
CPT utilization	Yes	237	58.66
	No	167	41.34
ART drug Adherence	Good adherence	226	55.94
	Fair/poor adherence	178	44.06
Disclosure status	Yes	255	63.12
	No	149	36.88
ART initiation time	Early	221	54.70
	Late	183	45.30
Baseline ART regimen	TDF/3TC/EFV	254	62.87
	TDF/3TC/DTG	111	27.48
	Others	39	9.65
ART drug side effect	Yes	43	10.64
	No	361	89.36
History of treatment failure	Yes	18	4.46
	No	386	95.54

*OIs* opportunistic infection other than TB, *CPT* cotrimoxazole preventive therapy, *IPT* isoniazid preventive therapy, *Others* include AZT/3TC/NVP ART regimen, TDF/3TC/NVP, ART regimen, AZT/3TC/EFV ART regimen…

### BMI changes status of the study participants

**Figure 1:**
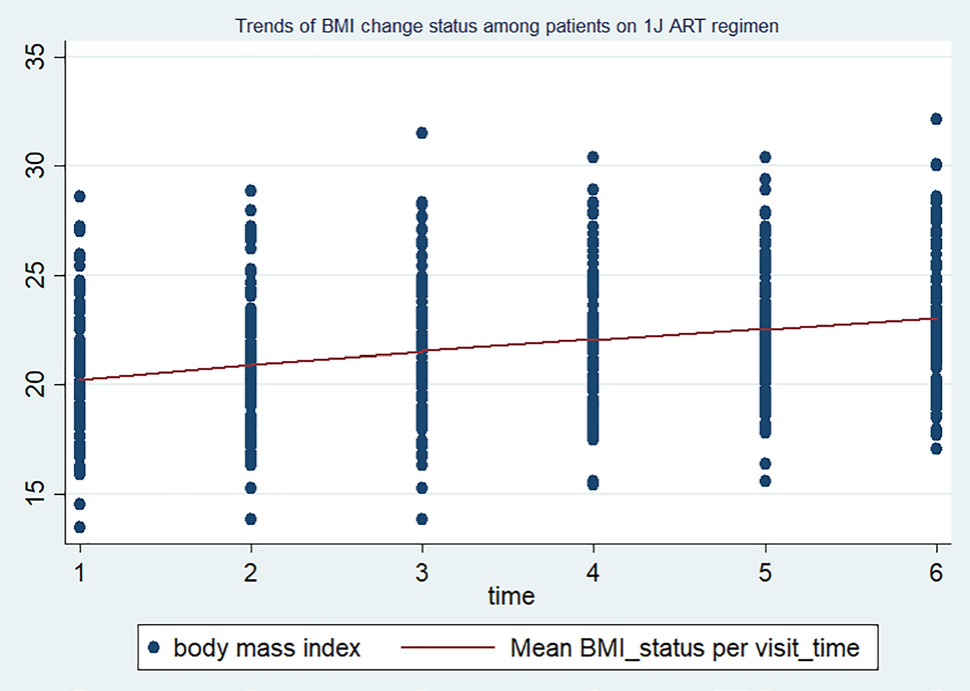
Trends of BMI changes over time among HIV-positive adults on TDF/3TC/DTG ART drug regimens in FHCSH, 2023.

**Figure 2:**
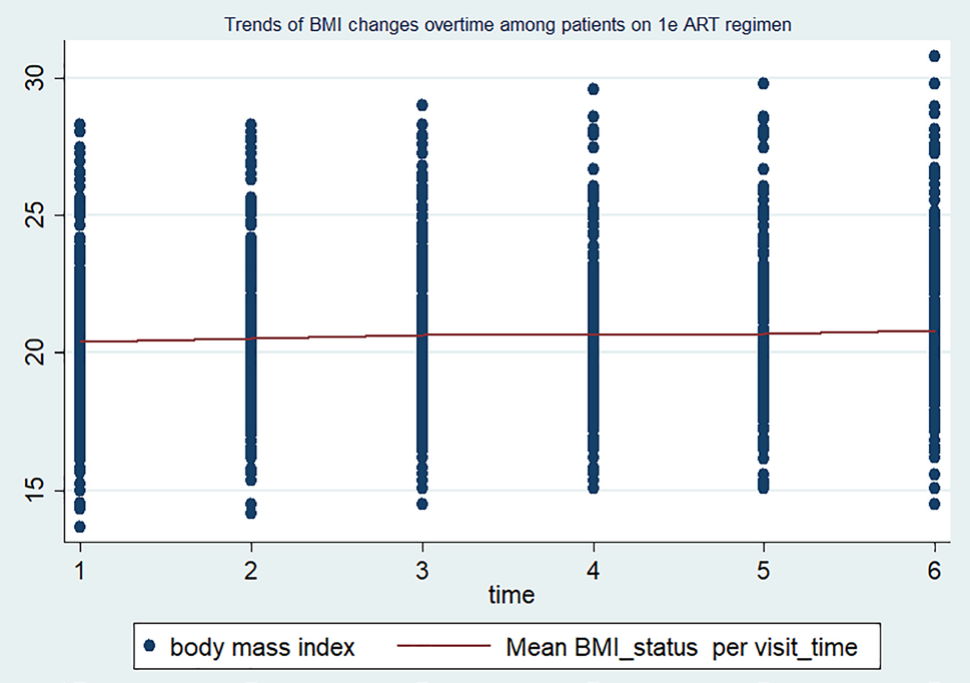
Trends of BMI changes over time among HIV-positive adults on TDF/3TC/EFV. ART drug regimens in FHCSH, 2023

**Figure 3:**
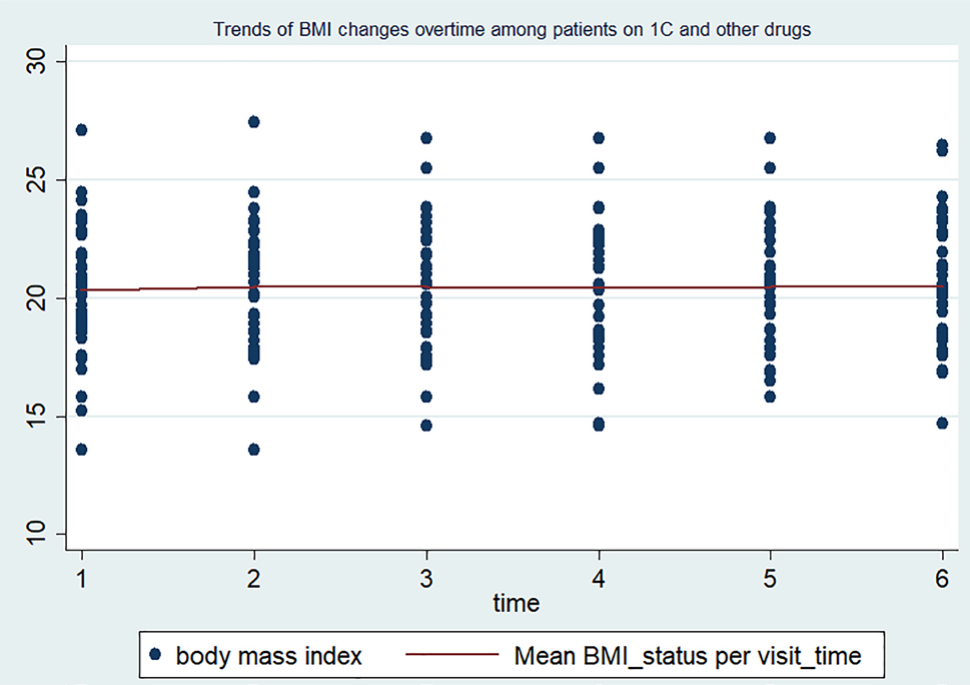
Trends of BMI changes over time among HIV-positive adults on other ART drug regimens in FHCSH, 2023.

The individuals were followed with a minimum of two up to a maximum of six follow-up schedules. The mean BMI status of the study participants in each visit was 20.34 kg/m^2^ (SD ± 2.8) at baseline, 20.64 kg/m^2^ (SD ± 2.7) at the 2nd visit, 20.9 kg/m^2^ (SD ± 2.8) at the 3rd visit, 21.0 kg/m^2^ (SD ± 2.8) at the 4th visit, 21.13 kg/m^2^ (SD ± 2.88) at the 5th visit, and 21.41 kg/m^2^ (SD ± 3.13) at the last visit, respectively. This indicates that during the total four-year follow-up period, the mean BMI status of the study participants increased from 20.34 kg/m^2^ (SD ± 2.8) to 21.41 kg/m^2^ (SD ± 3.13). At baseline, a total of 106 (26.3%) patients were underweight (BMI < 18.5 kg/m^2^) and 6.44% (26) of them were overweight (BMI > 25 kg/m^2^). Besides, at the compilation of their entire follow-up time, total of 73 (18.63%) patients were underweight (BMI < 18.5 kg/m^2^) and 7.7% (31) of them were overweight (BMI > 25 kg/m^2^), respectively. In the current study, the mean BMI level of men and women HIV-positive individuals changed from 19.56 kg/m^2^ (SD ± 2.74) to 22.67 kg/m^2^ (SD ± 3.23) and from 20.44 kg/m^2^ (SD ± 2.50) to 20.10 kg/m^2^ (SD ± 2.55), respectively. Correspondingly, almost all (183, 94%) men’s and less than half (57, 27.1%) of women showed BMI increments. Indeed, among male HIV-positive individuals, the BMI change was higher on those who are on the TDF/3TC/DTG ART regimen (increased from 20.09 to 23.73 kg/m^2^) than on those who are on the TDF/3TC/EFV ART drug regimen (increased from 20.12 to 21.82 kg/m^2^). Furthermore, the BMI increment is also high (95%) among HIV-positive adults aged 15–24 compared to the other age groups, where it increased from 19.58 kg/m^2^ (SD ± 3.10) to 23.03 kg/m^2^ (SD ± 3.59). Regarding the relationship between BMI changes and ART-drug regimens, it has been observed that HIV-positive adults on the TDF/3TC/DTG regimen experience a considerable increase in mean BMI levels. Specifically, their BMI levels increased from 20.28 kg/m^2^ (SD ± 2.80) to 23.05 kg/m^2^ (SD ± 3.27). On the other hand, individuals on the TDF/3TC/EFV drug regimen exhibit only a slight change in BMI levels, with an increase from 20.28 kg/m^2^ (SD ± 2.76) to 20.80 kg/m^2^ (SD ± 2.86). In line with the above context, the trends of BMI changes among individuals on the TDF/3TC/DTG ART regimen follow a linear increasing pattern, whereas those on the TDF/3TC/EFV and other ART drug regimens show steady and non-incremental patterns, respectively (Figs. 1, 2, and 3).

**Figure 4:**
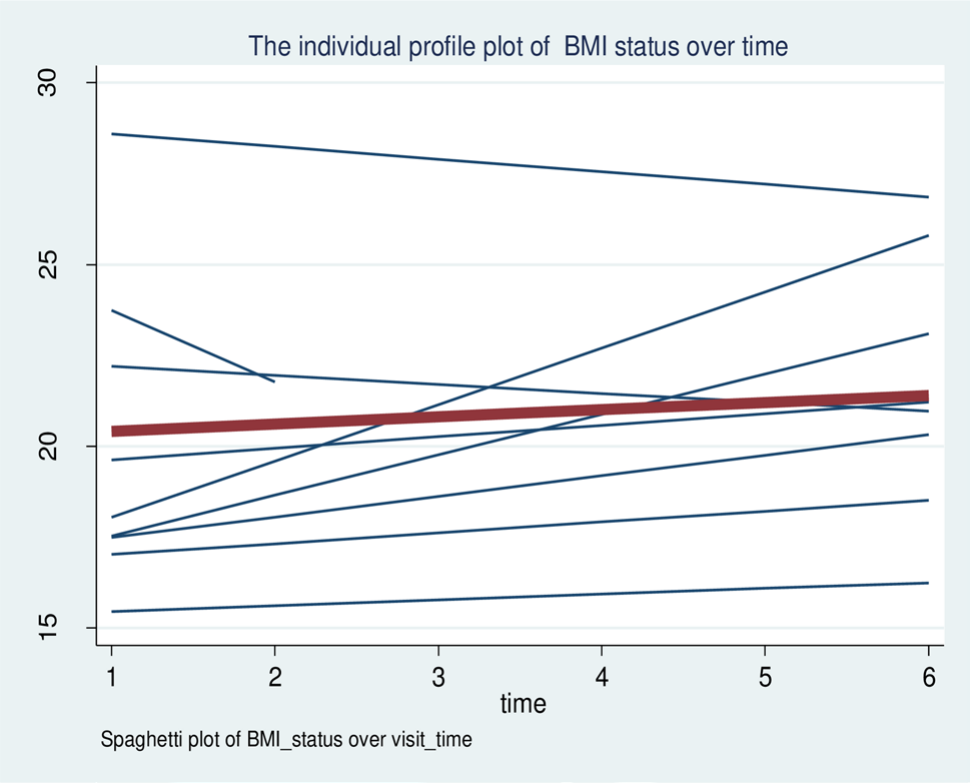
The individual profile plot of the BMI change status of HIV-positive adults receiving ART in FHCSH, 2023.

**Figure 5:**
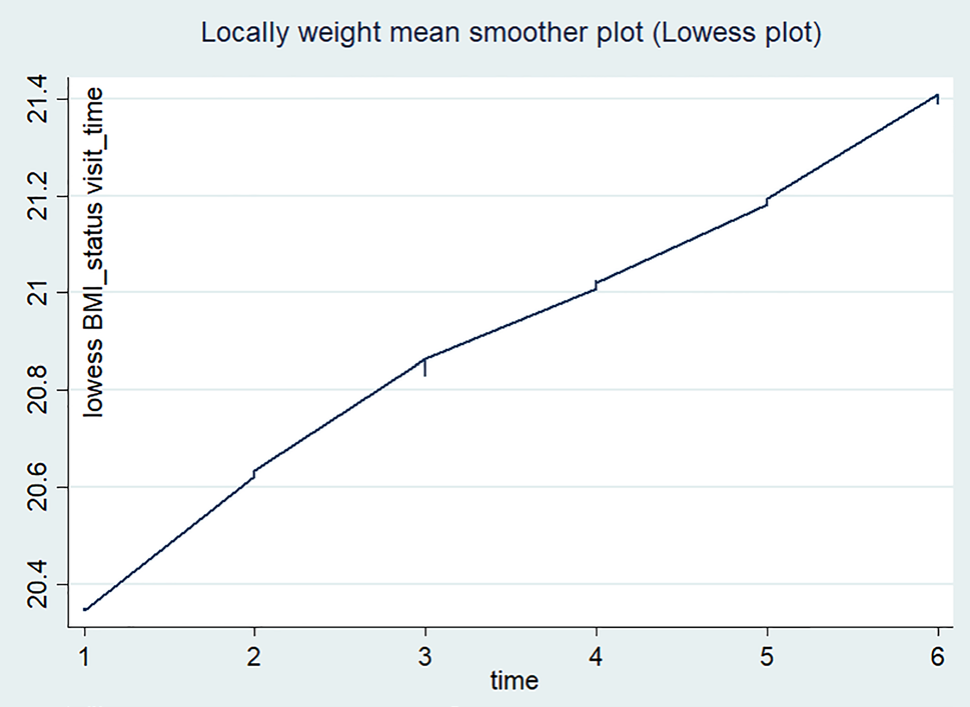
Locally weighted scatter plot smoother of BMI changes over time among adults receiving ART in FHCSH, 2023.

Over all, the individual profile plot and locally weighted mean smother plot were also used to navigate the BMI change status over time among adult HIV-positive individuals receiving ART in FHCSH. The individual profile plots of 50 participants indicated that there is great variability in BMI changes across individuals (Fig. 4). Moreover, the mean smoother plot showed that the BMI status of the individuals increased with a linear trend over time, which ensures the use of Generalized Estimated Equation method of analysis (Fig. 5).

### Predictors of BMI changes status of study participants

Twelve variables with a p-value of less than 0.25 were selected to be in the final model during the bi-variable GEE analysis. In the multivariable GEE model, the following factors were found to be significant predictors of changes in BMI level over time: duration of ART follow-up, employment status, WHO clinical staging, baseline ART regimen, and the interaction of ART follow-up duration with baseline ART regimen, WHO clinical staging, and TB/HIV co-infection.

As the ART follow-up number increases by one unit, the mean BMI level increases by 0.2 kg/m^2^ (β = 0.20, 95% CI 0.16 to 0.24). The change in BMI level over time in HIV-positive adults with non-employed occupational status is 0.96 kg/m^2^ (β = − 0.96, 95% CI − 1.67 to − 0.25) lower than in their counterparts. In comparison to WHO stage I or II clinical diseases, WHO stage III/IV clinical diseases lower the average BMI level of HIV-positive adults by 0.92 kg/m^2^ (β = − 0.92, 95% CI − 1.57 to − 0.35). When compared with patients on the TDF/3TC/EFVART drug regimen, being on the TDF/3TC/DTG regimen increased the average BMI level of the study participants by 0.95 kg/m^2^ (β = 0.95, 95% CI 0.32 to 1.57). Similarly, the interaction effect ART of follow-up time with TDF/3TC/DTG ART drug regimen increases the average BMI status of HIV-positive adults by 2.16 kg/m^2^ (β = 2.16, 95% CI 1.84 to 2.84) Even though there is no statistically significant difference in the average BMI change between those with and without TB/HIV co-infection, the interaction effect with time suggests that, as follow-up times increase, there may be a higher BMI increment among individuals without TB/HIV co-infection (Table 4).

**Table 4:** Multivariable generalized estimated equation analysis to identify predictors of BMI variation over time among HIV-positive adults receiving ART in Felegehiwot comprehensive specialized hospital, Northern Ethiopia, 2023 (N = 404).

Variables	Category	β-Coefficient (95% CI)	p value
Intercept		20.77 (19.43 to 22.11)	< **0.01**
ART follow-up duration		0.19 (0.161 to 0.21)	**< 0.01**
Sex	Female	Ref	
	Male	0.46 (− 0.19 to 1.11)	0.163
Educational status	Not have formal education	Ref	
	Primary	− 0.33 (− 1.03 to 0.37)	0.355
	Secondary	− 0.02 (− 0.76 to 0.71)	0.944
	Tertiary	− 0.05 (− 1.01 to 0.90)	0.917
Marital status	Married	Ref	Ref
	Not married	− 0.18 (− 0.72 to 0.362)	0.52
Employment status	Employed	Ref	
	Non-employed	− 0.96 (− 1.67 to − 0.254)	**< 0.01**
HIV disclosure status	Yes	Ref	Ref
	No	0.384 (− 0.22 to 0.99)	0.215
TB/HIV co-infection	Yes	Ref	Ref
	No	0.21 (− 0.55 to 0.97)	0.589
Baseline CD4 level	> 500 cell/mm^3^	Ref	Ref
	200–500 cell/mm^3^	0.92 (0.30 to 1.54)	0.770
	< 200 cells/mm^3^	− 0.09 (− 0.96 to 0.77)	0.831
WHO clinical staging	WHO stage I/II	Ref	Ref
	WHO stage III/IV	− 0.92 (− 1.53 to − 0.31)	**0.01**
Functional status	Working	Ref	Ref
	Ambulatory/Bedridden	− 0.44 (− 1.07 to 0.189)	0.170
IPT utilization	Yes	Ref	Ref
	No	0.11 (− 0.49 to 0.71)	0.731
Baseline ART regimen	TDF/3TC/EFV	Ref	Ref
	TDF/3TC/DTG	0.92 (0.30 to 1.54)	**0.01**
	Others	0.01 (− 0.84 to 0.87)	0.761
TB/HIV co-infection * follow-up duration	Yes	Ref	Ref
	No	1.89 (1.57 to 2.87)	**0.01**
Functional status * follow-up duration	Working	Ref	Ref
	Ambulatory/bedridden	0.01 (− 0.33 to 0.33)	0.95
Baseline ART * follow-up duration	TDF/3TC/EFV	Ref	Ref
	TDF/3TC/DTG	2.16 (1.84 to 2.48)	**0.01**
	Others	− 0.26 (− 0.74 to 0.23	0.29
WHO clinical staging * follow-up duration	WHO stage I/II	Ref	Ref
	WHO stage III/IV	− 1.43 (− 1.71 to − 1.15)	**0.01**

*3TC* lamivudine, *CI* confidence interval, *DTG* Dolutegravir, *IPT* isoniazid preventive therapy, *Ref* reference category, *TB* tuberculosis, *TDF* tenofovir, *WHO* World Health Organization, *Others* AZT/3TC/EFV, AZT/3TC/NVP, TDF/3TC/NVP ART regimens.

Variables significantly associated in the multivariable generelized equation model are in bold.

## Discussion

In this longitudinal study, the variations in BMI over time follow a linearly increasing trend. In this regard, it was observed that there was a clear association between the length of ART follow-up time and the change in BMI level of HIV-positive adults. The average BMI level is increased by 0.2 kg/m^2^ for a one-unit increase in the ART follow -up number of HIV-positive adults. This finding is consistent with prior related studies conducted in Ethiopia^32–35^, Tanzania^36^, South Africa^37,38^, and United States America^39^. The increase in BMI level over time in patients who are on ART could be due to the reduction in the metabolic demand of body cells^40^, the reversal of HIV-associated catabolism^41^, the return of gastrointestinal function, the decreased incidence of opportunistic infections^20,21^ and the redistribution of fat as a component of Lipodystrophy^42,43^. This implies that the longer HIV-positive adults are on ART, the better nutritional status and survival of patients.

Compared to the employed, the average BMI level of the non-employed HIV-positive adults decreased by 0.96 kg/m^2^. The result is in line with related literature’s undertaken in southern Ethiopia, central Ethiopia, and a meta-analysis report conducted in Sub-Saharan Africa^2,30,31^. The reason for the observed association could be explained by the fact that unemployed HIV-positive individuals are particularly prone to household food insecurity, which in turn increases the level of undernutrition. Furthermore, HIV-positive adults who were unemployed had poor adherence to ART, which increased the likelihood of acquiring opportunistic infections and viral replications^44^. This finding further suggests that increasing the socio-economic status of HIV-positive adults needs to be incorporated as an integral part of comprehensive HIV care.

The change in BMI status in HIV-positive adults is higher among individuals who are on the new TDF/3TC/DTG ART drug regimens compared to the previous TDF/3TC/EFV ART regimen. HIV-positive adults who are on the IJ ART regimen had almost a parallel baseline BMI status and showed a higher increment over their follow-up period than those individuals on the 1E ART regimen. This finding is supported by many observational and experimental studies conducted in various geographical settings around the world^20,22,45–49^. ART drug regimens containing integrase strand transfer inhibitors (INSTIs), particularly DTG, have been reported to have the highest weight gain in these HIV-positive patients, with an average weight gain ranging from 2.4 kg up to 6 kg after one year in low-income settings^20,49,50^. The possible reason for the observed link is that the newly endorsed Dolutegravir based ART regimen TDF/3TC/DTG is known to have rapid viral suppression effect^45,51^, which in turn decreases the energy expenditure to fight infections. Additionally, the TDF/3TC/DTG ART drug regimen is well tolerated and prepared in a single and fixed-dose combination formula that hinders the discontinuation of the intake of ART drugs^52,53^.

The BMI increment status of HIV-positive adults with advanced HIV stages is low compared to patients with WHO stages I or II clinical illnesses. The finding is supported by prior studies conducted in Ethiopia^25,32,34,54^, Zimbabwe^55^, Nepal^56^ and USA^40^. The decline in BMI status might be due to the presence of high nutritional requirements or calorie expenditures and the poor appetite and absorption of nutrients in individuals with advanced-stage clinical disease^4^. Indeed, advanced opportunistic infections such as chronic diarrhoea, fever, oral, and esophageal candidiasis are common features of WHO III/IV clinical disease, which all lead to decreased BMI levels, poor nutrition, and finally death^4,27^. This suggests that as the disease stage progresses, the likelihood of having a low BMI status increases, necessitating a high priority be placed on nutritional counseling and management. This could be explained by the fact that individuals with TB/HIV co-infection might experience more severe symptoms and complications, including decreased appetite, higher metabolic demand, difficulty adhering to both ART and a healthy diet, and subsequently a lower BMI status^10^. The above finding is in line with prior studies conducted in Ethiopia^25,57^, Kenya^58^, Guinea^59^ and Brazil^60^.

### Strength and limitation of the study

In order to handle the correlation between repeated measures and obtain valid estimates of predictors of trends and predictors of BMI changes, a generalised estimating equation (GEE) model of analysis was fitted. This study also has some limitations. Initially, due to the secondary nature of the data, it was hard to assess the effect of some predictor variables like viral load, dietary intake, household food security status, substance abuse status, the presence of chronic diseases, and micronutrient levels. Secondly, recording bias might be present since we used the patient's medical record as a full source of information.

## Conclusion and recommendation

In the cohort, both the baseline and follow-up mean BMI levels of the participants fell in the normal range and increased with a linear trend. Duration of ART follow-up, employment status, type of ART regimen, WHO clinical staging, and the interaction effect of follow-up duration * baseline ART regimen, follow-up duration * WHO clinical staging, and follow-up duration * TB/HIV co-infections were identified as significant predictors of change in BMI status over time. Due attention and special consideration need to be given to those individual’s with advanced HIV/AIDS diseases and other identified risk groups.

## Data Availability

Data are available upon reasonable request. Data used to draw the conclusion of this study available upon request of the corresponding author.

## Abbreviations

AIC: Akakie Information Criteria
BMI: Body Mass Index
CPT: Cotrimoxazole preventive therapy
FHCSH: Felegehiwot Comprehensive Specialized Hospital
FMOH: Federal Ministry of Health in Ethiopia
GEE: Generalized estimating equations
IPT: Isoniazid preventive therapy
MRN: Medical record number
OIs: Opportunistic infections
QIC: Quasi-likelihood independence model criterion
WHO: World Health Organization
